# Thermodynamic Optimization for an Endoreversible Dual-Miller Cycle (DMC) with Finite Speed of Piston

**DOI:** 10.3390/e20030165

**Published:** 2018-03-05

**Authors:** Zhixiang Wu, Lingen Chen, Huijun Feng

**Affiliations:** 1 Institute of Thermal Science and Power Engineering, Naval University of Engineering, Wuhan 430033, China; 2 Military Key Laboratory for Naval Ship Power Engineering, Naval University of Engineering, Wuhan 430033, China; 3 College of Power Engineering, Naval University of Engineering, Wuhan 430033, China

**Keywords:** finite time thermodynamics, finite speed thermodynamics, Dual-Miller cycle, finite speed of piston, power output, thermal efficiency, ecological function

## Abstract

Power output (P), thermal efficiency (η) and ecological function (E) characteristics of an endoreversible Dual-Miller cycle (DMC) with finite speed of the piston and finite rate of heat transfer are investigated by applying finite time thermodynamic (FTT) theory. The parameter expressions of the non-dimensional power output ($\bar{P}$), η and non-dimensional ecological function ($\bar{E}$) are derived. The relationships between $\bar{P}$ and cut-off ratio (ρ), between $\bar{P}$ and η, as well as between $\bar{E}$ and ρ are demonstrated. The influences of ρ and piston speeds in different processes on $\bar{P}$, η and $\bar{E}$ are investigated. The results show that $\bar{P}$ and $\bar{E}$ first increase and then start to decrease with increasing ρ. The optimal cut-off ratio $\rho_{opt}$ will increase if piston speeds increase in heat addition processes and heat rejection processes. As piston speeds in different processes increase, the maximum values of $\bar{P}$ and $\bar{E}$ increase. The results include the performance characteristics of various simplified cycles of DMC, such as Otto cycle, Diesel cycle, Dual cycle, Otto-Atkinson cycle, Diesel-Atkinson cycle, Dual-Atkinson cycle, Otto-Miller cycle and Diesel-Miller cycle. Comparing performance characteristics of the DMC with different optimization objectives, when choosing $\bar{E}$ as optimization objective, η improves 26.4% compared to choosing $\bar{P}$ as optimization objective, while $\bar{P}$ improves 74.3% compared to choosing η as optimization objective. Thus, optimizing E is the best compromise between optimizing P and optimizing η. The results obtained can provide theoretical guidance to design practical DMC engines.

## 1. Introduction

Finite time thermodynamics (FTT) theory [1,2,3,4,5,6,7,8,9,10,11] plays an increasingly important role in analyzing and optimizing performance characteristics of the thermodynamic processes [12,13,14] and cycles [15,16,17,18,19]. With the development of FTT, its research objects extended from conventional heat engine [20,21,22], refrigerator and heat pump [23] to unconventional systems, such as chemically driven engine [24], quantum engine [25,26,27,28,29,30,31] and energy selective electron engine [32,33,34]. Until now, scholars have performed many FTT studies for internal combustion engine (ICE) cycles [35]. Analyses of the different optimization objective functions are also important work for studying performance characteristics of the ICE cycles. Some new optimization objective functions on the basis of the power output (P) and thermal efficiency (η), such as the specific power [36,37], power density [38,39], exergetic performance [40,41,42,43] and finite time exergoeconomic performance [44,45] had been proposed. Besides, Angulo-Brown [46] first introduced the ecological function $E^{\prime }=P-T_{L}\sigma$ for the heat engine cycle (HEC), where $T_{L}$ is the temperature of the cold reservoir and σ is the entropy generation rate of the HEC, $T_{L}\sigma$ means the power dissipation of the HEC, the definition ignored the difference between exergy and energy, Yan [47] made a modification later, $E=P-T_{0}\sigma$, where $T_{0}$ is the environment temperature, $T_{0}\sigma$ means the exergy loss of the HEC. Chen et al. [48] finally presented a unified exergy-based ecological function. Ecological function optimizations have been performed since then [49,50,51]. Angulo-Brown et al. [52] firstly applied E to analyze and optimize the performance for an irreversible Otto cycle (OC) with friction loss. Ust et al. [53] took E as a criterion to optimize the performance of an endoreversible regenerative Brayton cycle. Based on Ref. [52], Ust et al. [54] introduced the ecological coefficient of performance (ECOP) into HEC, and performed an analysis for an irreversible Carnot cycle. Barranco-Jiménez and Angulo-Brown [55] analyzed the performance of an endoreversible Curzon and Ahlborn engine based on maximum power out and maximum ecological criteria. After these, Moscato and Oliveira [56] optimized E and ECOP characteristics for an irreversible OC. Gonca and Sahin [57] performed an optimization for an air-standard irreversible Dual-Atkinson cycle (DAC) by taking account of finite rate of heat transfer (HT), heat leakage (HL) and internal irreversibilities based on E and ECOP criteria. Long and Liu [58] analyzed and optimized η and its boundary based on E criterion for a general cycle with considering non-isothermal HT process and internal dissipation. Chen et al. [59] studied the performance of an universal cycle based on P, η, σ and E criteria. Ge [60] analyzed and compared the influences of variable specific heat of working fluid on P, η and E for ICE cycles. In addition to above heat engine cycles, the ecological performance of conventional refrigerator [61,62,63] and unconventional engines, such as quantum [64], thermoacoustic [65], chemical [66], macro/nano thermosize [67], light-driven [68], energy selective electron [69], electro-chemical [70] and n-Müser [71] engines, have been investigated.

All studies of HECs mentioned above were based on the assumption that the times of adiabatic processes are small or negligible, and the temperatures of heat absorption and heat releasing processes vary with constant rates [72,73]. Agrawal and Menon [74] and Agrawal [75] investigated the effects of finite speed of the piston on work and P for reversible [74] and endoreversible [75] Carnot cycle. Petrescu et al. [76,77,78,79] performed analysis and optimization for irreversible OC [76], Diesel cycle (DC) [77] and Carnot cycle [78,79] based on P and η characteristics by applying finite speed thermodynamics (FST) and the direct method [80,81,82]. Yang et al. [83] optimized speed ratio of the piston and obtained the optimal heating load for an endoreversible finite speed Carnot heat pump cycle. Feng et al. [84,85] and Chen et al. [86] performed an analysis for optimal piston speed ratio and derived the analytical relationship between P and η for an endoreversible Carnot cycle with finite rate of HT [84], irreversible Carnot cycle with HL and irreversibility [85], as well as irreversible Carnot refrigerator and Carnot heat pump [86] by using FST and the direct method. Hosseinzade et al. [87] and Ahmadi et al. [88] investigated analysis and optimization for an irreversible Stirling cycle by applying FST and the direct method.

Some scholars have also performed thermodynamic optimization for Dual-Miller cycle (DMC) engines. Gonca et al. [89] carried out a study on P and η for an air-standard irreversible DMC with internal irreversibilities. Ust et al. [90] took internal and external irreversibilities into account to optimize the exergy output and exergetic performance coefficient of an irreversible DMC. Gonca et al. [91] examined and analyzed the effects of HT on P and η for an irreversible DMC with HT loss and internal irreversibilities. Gonca [92] considered internal irreversibilities to perform an optimization study on Diesel-Miller cycle (DiMC), Otto-Miller cycle (OMC) and DMC using the maximum power output and power density criteria, as well as the maximum thermal efficiency. Gonca and Sahin [93] considered finite rate of HT, HL and internal irreversibilities to analyze and optimize ECOP of irreversible ICE cycles, such as Dual-Diesel cycle (DDC), OMC and DMC. Wu et al. [94,95,96] established air-standard irreversible DMC models with constant specific heat [94], linear variable specific heat ratio [95] and nonlinear variable specific heat ratio [95] of working fluid, respectively, analyzed and optimized P, η and E of the cycles. You et al. [97] replaced the two reversible adiabatic processes of the DMC with two polytropic processes, studied P, η and E of the cycle.

DMC can be simplified to other gas cycles; the performance characteristics of other gas cycles are special cases of those of DMC. Based on the work mentioned above, this paper establishes an endoreversible DMC model with finite speed of the piston and finite rate of HT. The power output, thermal efficiency and ecological function are investigated based on assuming piston speeds are unequal constants in different processes by combining with FTT and FST [75,85]. The parameter expressions of non-dimensional power output, thermal efficiency and non-dimensional ecological function are derived. The effects of design parameter and piston speeds on the performance of the cycle are analyzed, and the impact degrees of the time in each process on the performance of the cycle are compared.

## 2. Cycle Model and Performance Analyses

**Figure 1:**
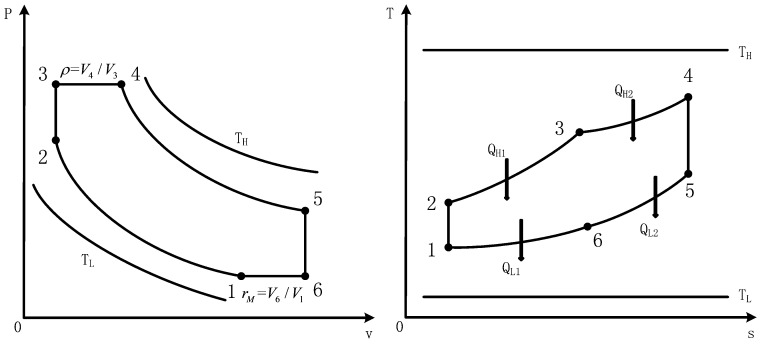
*P*-*v* and *T*-*s* diagrams for an endoreversible Dual-Miller cycle.

Figure 1 shows *P*-*v* and *T*-*s* diagrams of an endoreversible DMC (1-2-3-4-5-6), which includes constant temperature heat source and constant temperature heat sink. Processes 1→2 and 4→5 are adiabatic compression and adiabatic expansion processes; 2→3 and 5→6 are constant volume processes; and 3→4 and 6→1 are constant pressure processes.

Assuming the model is an air-standard cycle and the working fluid is an ideal gas. The working fluid absorbs heat in 2→3 and 3→4 processes, and releases heat in 5→6 and 6→1 processes. In the processes of 2→3 and 3→4, the quantities of heat provided by heat source $T_{H}$ are $Q_{H1}$ and $Q_{H2}$, respectively. In the processes of 5→6 and 6→1, the quantities of heat released to heat sink $T_{L}$ are $Q_{L1}$ and $Q_{L2}$, respectively. According to Refs. [68,90,91], one can assume that the law of HT obeys q∝(ΔT), there are

(1) $$Q_{H1}=U_{H1}F_{H1}\frac{(T_{H}-T_{2})-\left(T_{H}-T_{3}\right)}{\ln\left[\left(T_{H}-T_{2}\right)/\left(T_{H}-T_{3}\right)\right]}t_{2-3}=mC_{v}(T_{3}-T_{2})=mC_{v}\varepsilon_{H1}(T_{H}-T_{2})$$

(2) $$Q_{H2}=U_{H2}F_{H2}\frac{(T_{H}-T_{3})-\left(T_{H}-T_{4}\right)}{\ln\left[\left(T_{H}-T_{3}\right)/\left(T_{H}-T_{4}\right)\right]}t_{3-4}=mC_{p}(T_{4}-T_{3})=mC_{p}\varepsilon_{H2}(T_{H}-T_{3})$$

(3) $$Q_{L1}=U_{L1}F_{L1}\frac{(T_{6}-T_{L})-\left(T_{1}-T_{L}\right)}{\ln\left[\left(T_{6}-T_{L}\right)/\left(T_{1}-T_{L}\right)\right]}t_{6-1}=mC_{p}(T_{6}-T_{1})=mC_{p}\varepsilon_{L1}(T_{6}-T_{L})$$

(4) $$Q_{L2}=U_{L2}F_{L2}\frac{(T_{5}-T_{L})-\left(T_{6}-T_{L}\right)}{\ln\left[\left(T_{5}-T_{L}\right)/\left(T_{6}-T_{L}\right)\right]}t_{5-6}=mC_{v}(T_{5}-T_{6})=mC_{v}\varepsilon_{L2}(T_{5}-T_{L})$$

where $U_{H1}$, $U_{H2}$ and $U_{L1}$, $U_{L2}$ are HT coefficients, $W/(m^{2}\cdot K)$; $F_{H1}$, $F_{H2}$ and $F_{L1}$, $F_{L2}$ are HT areas of the heat exchangers between the working fluid and the heat reservoir, $m^{2}$; $t_{2-3}$, $t_{3-4}$, $t_{5-6}$ and $t_{6-1}$ are the times in processes 2→3, 3→4, 5→6 and 6→1, s; m is mass of working fluid, kg; $C_{v}$ and $C_{p}$ are specific heats at constant volume and constant pressure, J/(kg⋅K), they can be taken as constants because the working fluid is the ideal gas; and $\varepsilon_{H1}$, $\varepsilon_{H2}$ and $\varepsilon_{L1}$, $\varepsilon_{L2}$ are the effectiveness of heat exchangers in hot side and cold side, and they are given as:

(5) $$\begin{array}{l} \varepsilon_{H1}=1-\exp(-N_{H1}),\;\varepsilon_{H2}=1-\exp(-N_{H2})\\ \varepsilon_{L1}=1-\exp(-N_{L1}),\;\varepsilon_{L2}=1-\exp(-N_{L2}) \end{array}$$

where $N_{H1}$, $N_{H2}$ and $N_{L1}$, $N_{L2}$ are numbers of heat exchangers HT unites in hot side and cold side, which are expressed as:

(6) $$\begin{array}{l} N_{H1}=U_{H1}F_{H1}/({\dot{m}}_{2-3}C_{v}),\;N_{H2}=U_{H2}F_{H2}/({\dot{m}}_{3-4}C_{p})\\ N_{L1}=U_{L1}F_{L1}/({\dot{m}}_{6-1}C_{p}),\;N_{L2}=U_{L2}F_{L2}/({\dot{m}}_{5-6}C_{v}) \end{array}$$

where ${\dot{m}}_{2-3}$, ${\dot{m}}_{3-4}$, ${\dot{m}}_{6-1}$ and ${\dot{m}}_{5-6}$ are mass flow rates of working fluid in processes 2→3, 3→4, 6→1 and 5→6, kg/s.

The total heat addition quantity $Q_{H}$ is

(7) $$Q_{H}=Q_{H1}+Q_{H2}$$

The total heat rejection quantity $Q_{L}$ is

(8) $$Q_{L}=Q_{L1}+Q_{L2}$$

From Equations (1)–(4), one has

(9) $$T_{1}=\varepsilon_{L1}T_{L}+(1-\varepsilon_{L1})\varepsilon_{L2}T_{L}+(1-\varepsilon_{L1})(1-\varepsilon_{L2})T_{5}$$

(10) $$T_{3}=\varepsilon_{H1}T_{H}+(1-\varepsilon_{H1})T_{2}$$

(11) $$T_{4}=\varepsilon_{H2}T_{H}+(1-\varepsilon_{H2})\varepsilon_{H1}T_{H}+(1-\varepsilon_{H1})(1-\varepsilon_{H2})T_{2}$$

(12) $$T_{6}=\varepsilon_{L2}T_{L}+(1-\varepsilon_{L2})T_{5}$$

The definitions of cut-off ratio and Miller cycle ratio are

(13) $$\rho =V_{4}/V_{3}=T_{4}/T_{3}$$

(14) $$r_{M}=V_{6}/V_{1}=T_{6}/T_{1}$$

The following equation is obtained based on the second law of thermodynamics [89]

(15) $$T_{1}^{k}T_{4}^{k}=T_{2}T_{5}T_{3}^{k-1}T_{6}^{k-1}$$

Combining Equations (10), (13) and (15), the function of $T_{2}$ as related with $T_{5}$ is derived. Combining Equations (12), (14) and (15), the function of $T_{5}$ as related with $T_{2}$ is derived.

(16) $$T_{2}=\frac{\varepsilon_{H1}T_{H}}{\rho^{-k}T_{1}{}^{-k}T_{5}T_{6}{}^{k-1}-(1-\varepsilon_{H1})}=f_{1}(T_{5})$$

(17) $$T_{5}=\frac{\varepsilon_{L2}T_{L}}{r_{M}{}^{k}T_{2}T_{3}{}^{k-1}T_{4}{}^{-k}-(1-\varepsilon_{L2})}=f_{2}(T_{2})$$

The work output W can be calculated as follows:

(18) $$\begin{array}{cc} W & =Q_{H}-Q_{L}\\ & =mC_{v}\left[\varepsilon_{H1}(T_{H}-T_{2})+k\varepsilon_{H2}(T_{H}-T_{3})-k\varepsilon_{L1}(T_{6}-T_{L})-\varepsilon_{L2}(T_{5}-T_{L})\right]\\ & =mC_{v}\left\{\left[k\varepsilon_{H2}(1-\varepsilon_{H1})+\varepsilon_{H1}\right](T_{H}-T_{2})-\left[k\varepsilon_{L1}(1-\varepsilon_{L2})+\varepsilon_{L2}\right](T_{5}-T_{L})\right\} \end{array}$$

Combining Equations (7) and (18), the thermal efficiency η is

(19) $$\begin{array}{cc} \eta =\frac{W}{Q_{H}} & =1-\frac{k\varepsilon_{L1}(T_{6}-T_{L})+\varepsilon_{L2}\left(T_{5}-T_{L}\right)}{\varepsilon_{H1}\left(T_{H}-T_{2}\right)+k\varepsilon_{H2}\left(T_{H}-T_{3}\right)}\\ & =1-\frac{\left[k\varepsilon_{L1}(1-\varepsilon_{L2})+\varepsilon_{L2}\right](T_{5}/T_{H}-T_{L}/T_{H})}{\left[k\varepsilon_{H2}(1-\varepsilon_{H1})+\varepsilon_{H1}\right](1-T_{2}/T_{H})} \end{array}$$

Considering the piston speed in each process is generally different, it can be assumed that the piston speeds in processes of 1-2, 2-3, 3-4, 4-5, 5-6, 6-1 are constants $u_{1-2}$, $u_{2-3}$, $u_{3-4}$, $u_{4-5}$, $u_{5-6}$, $u_{6-1}$, respectively, and $u_{1-2}=u_{4-5}$, $x=u_{1-2}/u_{3-4}$, $y=u_{1-2}/u_{6-1}$. Consequently, the times ($t_{1-2}$, $t_{2-3}$, $t_{3-4}$, $t_{4-5}$, $t_{5-6}$, $t_{6-1}$) in six processes can be expressed as follows:

(20) $$t_{1-2}=\frac{V_{1}-V_{2}}{A_{ps}u_{1-2}}=\frac{V_{2}}{A_{ps}u_{1-2}}(\frac{V_{1}}{V_{2}}-1)=\frac{L_{2}}{u_{1-2}}[{\left(\frac{T_{2}}{T_{1}}\right)}^{\frac{1}{k-1}}-1]$$

(21) $$t_{3-4}=\frac{V_{4}-V_{3}}{A_{ps}u_{3-4}}=\frac{V_{3}}{A_{ps}u_{3-4}}(\frac{V_{4}}{V_{3}}-1)=\frac{V_{2}}{A_{ps}u_{1-2}}\frac{u_{1-2}}{u_{3-4}}(\frac{V_{4}}{V_{3}}-1)=\frac{L_{2}}{u_{1-2}}x(\frac{T_{4}}{T_{3}}-1)$$

(22) $$t_{4-5}=\frac{V_{5}-V_{4}}{A_{ps}u_{4-5}}=\frac{V_{4}}{A_{ps}u_{1-2}}(\frac{V_{5}}{V_{4}}-1)=\frac{V_{2}}{A_{ps}u_{1-2}}\frac{V_{4}}{V_{3}}(\frac{V_{5}}{V_{4}}-1)=\frac{L_{2}}{u_{1-2}}\frac{T_{4}}{T_{3}}[{\left(\frac{T_{4}}{T_{5}}\right)}^{\frac{1}{k-1}}-1]$$

(23) $$t_{6-1}=\frac{V_{6}-V_{1}}{A_{ps}u_{6-1}}=\frac{V_{1}}{A_{ps}u_{6-1}}(\frac{V_{6}}{V_{1}}-1)=\frac{V_{2}}{A_{ps}u_{1-2}}\frac{u_{1-2}}{u_{6-1}}\frac{V_{1}}{V_{2}}(\frac{V_{6}}{V_{1}}-1)=\frac{L_{2}}{u_{1-2}}y{\left(\frac{T_{2}}{T_{1}}\right)}^{\frac{1}{k-1}}(\frac{T_{6}}{T_{1}}-1)$$

(24) $$t_{2-3}=a\cdot t_{3-4}$$

(25) $$t_{5-6}=b\cdot t_{6-1}$$

where $A_{ps}$ is cross section area of piston, $m^{2}$; $L_{2}=V_{2}/A_{ps}$, m; a and b are constants.

Combining Equations (9)–(12), the cycle period τ is

(26) $$\begin{array}{cc} \tau = & t_{1-2}+t_{2-3}+t_{3-4}+t_{4-5}+t_{5-6}+t_{6-1}\\ = & \frac{L_{2}}{u_{1-2}}\{{\left\{\left(1-\varepsilon_{L1})(1-\varepsilon_{L2})(T_{5}/T_{2})+\left[(1-\varepsilon_{L1})\varepsilon_{L2}+\varepsilon_{L1}\right](T_{L}/T_{2}\right)\right\}}^{k-1}\cdot \{(1+b)y[\left(1-\varepsilon_{L1}\right)\\ & {+\frac{\varepsilon_{L1}}{\varepsilon_{L2}+(1-\varepsilon_{L2})\cdot {\left(T_{L}/T_{5}\right)}^{-1}}]}^{-1}-(1+b)y+1\}+\left[(1-\varepsilon_{H2})+\frac{\varepsilon_{H2}}{\varepsilon_{H1}+(1-\varepsilon_{H1})(T_{2}/T_{H})}\right]\\ & \times \left\{{\left\{(1-\varepsilon_{H1})(1-\varepsilon_{H2})\cdot {\left(T_{5}/T_{2}\right)}^{-1}+\left[(1-\varepsilon_{H2})\varepsilon_{H1}+\varepsilon_{H2}\right]\cdot {\left(T_{5}/T_{H}\right)}^{-1}\right\}}^{\frac{1}{k-1}}+(1+a)x-1\right\}\\ & -\left[(1+a)x+1\right]\} \end{array}$$

The power output P is

(27) P=W/τ

The non-dimensional power output $\bar{P}$ can be obtained as:

(28) $$\begin{array}{cc} \bar{P}= & \frac{P}{mC_{v}T_{H}}\frac{L_{2}}{u_{1-2}}\\ = & \frac{\left[k\varepsilon_{H2}(1-\varepsilon_{H1})+\varepsilon_{H1}\right](1-T_{2}/T_{H})-\left[k\varepsilon_{L1}(1-\varepsilon_{L2})+\varepsilon_{L2}\right](T_{5}/T_{H}-T_{L}/T_{H})}{\begin{array}{l} {\left\{\left(1-\varepsilon_{L1})(1-\varepsilon_{L2})(T_{5}/T_{2})+\left[(1-\varepsilon_{L1})\varepsilon_{L2}+\varepsilon_{L1}\right](T_{L}/T_{2}\right)\right\}}^{k-1}\cdot \{(1+b)y[\left(1-\varepsilon_{L1}\right)\\ {+\frac{\varepsilon_{L1}}{\varepsilon_{L2}+(1-\varepsilon_{L2})\cdot {\left(T_{L}/T_{5}\right)}^{-1}}]}^{-1}-(1+b)y+1\}+\left[(1-\varepsilon_{H2})+\frac{\varepsilon_{H2}}{\varepsilon_{H1}+(1-\varepsilon_{H1})(T_{2}/T_{H})}\right]\\ \times \left\{{\left\{(1-\varepsilon_{H1})(1-\varepsilon_{H2})\cdot {\left(T_{5}/T_{2}\right)}^{-1}+\left[(1-\varepsilon_{H2})\varepsilon_{H1}+\varepsilon_{H2}\right]\cdot {\left(T_{5}/T_{H}\right)}^{-1}\right\}}^{\frac{1}{k-1}}+(1+a)x-1\right\}\\ -\left[(1+a)x+1\right] \end{array}} \end{array}$$

The entropy generation ΔS can be written as follows:

(29) $$\begin{array}{cc} \Delta S=\frac{Q_{L}}{T_{L}}-\frac{Q_{H}}{T_{H}} & =\frac{mC_{v}\left[k\varepsilon_{L1}(T_{6}-T_{L})+\varepsilon_{L2}(T_{5}-T_{L})\right]}{T_{L}}-\frac{mC_{v}\left[\varepsilon_{H1}(T_{H}-T_{2})+k\varepsilon_{H2}(T_{H}-T_{3})\right]}{T_{H}}\\ & =mC_{v}\left\{\left[k\varepsilon_{L1}(1-\varepsilon_{L2})+\varepsilon_{L2}\right](T_{5}/T_{L}-1)-\left[k\varepsilon_{H2}(1-\varepsilon_{H1})+\varepsilon_{H1}\right](1-T_{2}/T_{H})\right\} \end{array}$$

Thus, the entropy generation rate σ is

(30) σ=ΔS/τ

The ecological function [46,47] is given as:

(31) $$E=P-T_{o}\sigma$$

The non-dimensional ecological function $\bar{E}$ can be obtained as:

(32) $$\begin{array}{cc} \bar{E}= & \bar{P}-\frac{T_{o}\sigma }{mC_{v}T_{H}}\frac{L_{2}}{u_{1-2}}\\ = & \frac{\begin{array}{l} \left[k\varepsilon_{H2}(1-\varepsilon_{H1})+\varepsilon_{H1}\right](1-T_{2}/T_{H})(1+T_{0}/T_{H})-\left[k\varepsilon_{L1}(1-\varepsilon_{L2})+\varepsilon_{L2}\right][\left(T_{5}/T_{H}-T_{L}/T_{H}\right)\\ +(T_{5}/T_{L}-1)(T_{0}/T_{H})] \end{array}}{\begin{array}{l} {\left\{\left(1-\varepsilon_{L1})(1-\varepsilon_{L2})(T_{5}/T_{2})+\left[(1-\varepsilon_{L1})\varepsilon_{L2}+\varepsilon_{L1}\right](T_{L}/T_{2}\right)\right\}}^{k-1}\cdot \{(1+b)y[\left(1-\varepsilon_{L1}\right)\\ {+\frac{\varepsilon_{L1}}{\varepsilon_{L2}+(1-\varepsilon_{L2})\cdot {\left(T_{L}/T_{5}\right)}^{-1}}]}^{-1}-(1+b)y+1\}+\left[(1-\varepsilon_{H2})+\frac{\varepsilon_{H2}}{\varepsilon_{H1}+(1-\varepsilon_{H1})(T_{2}/T_{H})}\right]\\ \times \left\{{\left\{(1-\varepsilon_{H1})(1-\varepsilon_{H2})\cdot {\left(T_{5}/T_{2}\right)}^{-1}+\left[(1-\varepsilon_{H2})\varepsilon_{H1}+\varepsilon_{H2}\right]\cdot {\left(T_{5}/T_{H}\right)}^{-1}\right\}}^{\frac{1}{k-1}}+(1+a)x-1\right\}\\ -\left[(1+a)x+1\right] \end{array}} \end{array}$$

## 3. Analyses of Special Cases

Equations (19), (28) and (32) are expressions of η, $\bar{P}$ and $\bar{E}$ of an endoreversible DMC. When the temperatures at different state points reach certain relationships, they can be transformed into expressions of η, $\bar{P}$ and $\bar{E}$ of different simplified cycles with finite speed of the piston.

(1) When $T_{2}=T_{3}$, i.e., $\varepsilon_{H1}=0$, Equations (19), (28) and (32) are transformed into expressions of η, $\bar{P}$ and $\bar{E}$ of an endoreversible DiMC with finite speed of the piston and finite rate of HT:
  (33) $$\eta_{DiMC}=1-\frac{\left[k\varepsilon_{L1}(1-\varepsilon_{L2})+\varepsilon_{L2}\right](T_{5}/T_{H}-T_{L}/T_{H})}{k\varepsilon_{H2}(1-T_{2}/T_{H})}$$
  (34) $${\bar{P}}_{DiMC}=\frac{k\varepsilon_{H2}(1-T_{2}/T_{H})-\left[k\varepsilon_{L1}(1-\varepsilon_{L2})+\varepsilon_{L2}\right](T_{5}/T_{H}-T_{L}/T_{H})}{\begin{array}{l} {\left\{\left(1-\varepsilon_{L1})(1-\varepsilon_{L2})(T_{5}/T_{2})+\left[(1-\varepsilon_{L1})\varepsilon_{L2}+\varepsilon_{L1}\right](T_{L}/T_{2}\right)\right\}}^{k-1}\cdot \{(1+b)y[\left(1-\varepsilon_{L1}\right)\\ {+\frac{\varepsilon_{L1}}{\varepsilon_{L2}+(1-\varepsilon_{L2})\cdot {\left(T_{L}/T_{5}\right)}^{-1}}]}^{-1}-(1+b)y+1\}+\left[(1-\varepsilon_{H2})+\frac{\varepsilon_{H2}}{\left(T_{2}/T_{H}\right)}\right]\\ \times \left\{{\left[(1-\varepsilon_{H2})\cdot {\left(T_{5}/T_{2}\right)}^{-1}+\varepsilon_{H2}\cdot {\left(T_{5}/T_{H}\right)}^{-1}\right]}^{\frac{1}{k-1}}+(1+a)x-1\right\}-\left[(1+a)x+1\right] \end{array}}$$
  (35) $${\bar{E}}_{DiMC}=\frac{\begin{array}{l} k\varepsilon_{H2}(1-T_{2}/T_{H})(1+T_{0}/T_{H})-\left[k\varepsilon_{L1}(1-\varepsilon_{L2})+\varepsilon_{L2}\right][\left(T_{5}/T_{H}-T_{L}/T_{H}\right)\\ +(T_{5}/T_{L}-1)(T_{0}/T_{H})] \end{array}}{\begin{array}{l} {\left\{\left(1-\varepsilon_{L1})(1-\varepsilon_{L2})(T_{5}/T_{2})+\left[(1-\varepsilon_{L1})\varepsilon_{L2}+\varepsilon_{L1}\right](T_{L}/T_{2}\right)\right\}}^{k-1}\cdot \{(1+b)y[\left(1-\varepsilon_{L1}\right)\\ {+\frac{\varepsilon_{L1}}{\varepsilon_{L2}+(1-\varepsilon_{L2})\cdot {\left(T_{L}/T_{5}\right)}^{-1}}]}^{-1}-(1+b)y+1\}+\left[(1-\varepsilon_{H2})+\frac{\varepsilon_{H2}}{\left(T_{2}/T_{H}\right)}\right]\\ \times \left\{{\left[(1-\varepsilon_{H2})\cdot {\left(T_{5}/T_{2}\right)}^{-1}+\varepsilon_{H2}\cdot {\left(T_{5}/T_{H}\right)}^{-1}\right]}^{\frac{1}{k-1}}+(1+a)x-1\right\}-\left[(1+a)x+1\right] \end{array}}$$
(2) When $T_{3}=T_{4}$, i.e., $\varepsilon_{H2}=0$, Equations (19), (28) and (32) are transformed into expressions of η, $\bar{P}$ and $\bar{E}$ of an endoreversible OMC with finite speed of the piston and finite rate of HT:
  (36) $$\eta_{OMC}=1-\frac{\left[k\varepsilon_{L1}(1-\varepsilon_{L2})+\varepsilon_{L2}\right](T_{5}/T_{H}-T_{L}/T_{H})}{\varepsilon_{H1}(1-T_{2}/T_{H})}$$
  (37) $${\bar{P}}_{OMC}=\frac{\varepsilon_{H1}(1-T_{2}/T_{H})-\left[k\varepsilon_{L1}(1-\varepsilon_{L2})+\varepsilon_{L2}\right](T_{5}/T_{H}-T_{L}/T_{H})}{\begin{array}{l} {\left\{\left(1-\varepsilon_{L1})(1-\varepsilon_{L2})(T_{5}/T_{2})+\left[(1-\varepsilon_{L1})\varepsilon_{L2}+\varepsilon_{L1}\right](T_{L}/T_{2}\right)\right\}}^{k-1}\cdot \{(1+b)y[\left(1-\varepsilon_{L1}\right)\\ {+\frac{\varepsilon_{L1}}{\varepsilon_{L2}+(1-\varepsilon_{L2})\cdot {\left(T_{L}/T_{5}\right)}^{-1}}]}^{-1}-(1+b)y+1\}+{\left[(1-\varepsilon_{H1})\cdot {\left(\frac{T_{5}}{T_{2}}\right)}^{-1}+\varepsilon_{H1}\cdot {\left(\frac{T_{5}}{T_{H}}\right)}^{-1}\right]}^{\frac{1}{k-1}}\\ -2 \end{array}}$$
  (38) $${\bar{E}}_{OMC}=\frac{\begin{array}{l} \varepsilon_{H1}(1-T_{2}/T_{H})(1+T_{0}/T_{H})-\left[k\varepsilon_{L1}(1-\varepsilon_{L2})+\varepsilon_{L2}\right][\left(T_{5}/T_{H}-T_{L}/T_{H}\right)\\ +(T_{5}/T_{L}-1)(T_{0}/T_{H})] \end{array}}{\begin{array}{l} {\left\{\left(1-\varepsilon_{L1})(1-\varepsilon_{L2})(T_{5}/T_{2})+\left[(1-\varepsilon_{L1})\varepsilon_{L2}+\varepsilon_{L1}\right](T_{L}/T_{2}\right)\right\}}^{k-1}\cdot \{(1+b)y[\left(1-\varepsilon_{L1}\right)\\ {+\frac{\varepsilon_{L1}}{\varepsilon_{L2}+(1-\varepsilon_{L2})\cdot {\left(T_{L}/T_{5}\right)}^{-1}}]}^{-1}-(1+b)y+1\}+{\left[(1-\varepsilon_{H1})\cdot {\left(\frac{T_{5}}{T_{2}}\right)}^{-1}+\varepsilon_{H1}\cdot {\left(\frac{T_{5}}{T_{H}}\right)}^{-1}\right]}^{\frac{1}{k-1}}\\ -2 \end{array}}$$
(3) When $T_{5}=T_{6}$, i.e., $\varepsilon_{L2}=0$, Equations (19), (28) and (32) are transformed into expressions of η, $\bar{P}$ and $\bar{E}$ of an endoreversible DAC with finite speed of the piston and finite rate of HT:
  (39) $$\eta_{DAC}=1-\frac{k\varepsilon_{L1}(T_{5}/T_{H}-T_{L}/T_{H})}{\left[k\varepsilon_{H2}(1-\varepsilon_{H1})+\varepsilon_{H1}\right](1-T_{2}/T_{H})}$$
  (40) $${\bar{P}}_{DAC}=\frac{\left[k\varepsilon_{H2}(1-\varepsilon_{H1})+\varepsilon_{H1}\right](1-T_{2}/T_{H})-k\varepsilon_{L1}(T_{5}/T_{H}-T_{L}/T_{H})}{\begin{array}{l} {\left[\left(1-\varepsilon_{L1})(T_{5}/T_{2})+\varepsilon_{L1}(T_{L}/T_{2}\right)\right]}^{k-1}\cdot \left\{(1+b)y{\left[\left(1-\varepsilon_{L1})+\varepsilon_{L1}(T_{L}/T_{5}\right)\right]}^{-1}-(1+b)y+1\right\}\\ +\left[(1-\varepsilon_{H2})+\frac{\varepsilon_{H2}}{\varepsilon_{H1}+(1-\varepsilon_{H1})(T_{2}/T_{H})}\right]\times \{\{(1-\varepsilon_{H1})(1-\varepsilon_{H2})\cdot {\left(\frac{T_{5}}{T_{2}}\right)}^{-1}+[(1-\varepsilon_{H2})\varepsilon_{H1}\\ {+\varepsilon_{H2}]\cdot {\left(T_{5}/T_{H}\right)}^{-1}\}}^{\frac{1}{k-1}}+(1+a)x-1\}-\left[(1+a)x+1\right] \end{array}}$$
  (41) $${\bar{E}}_{DAC}=\frac{\begin{array}{l} \left[k\varepsilon_{H2}(1-\varepsilon_{H1})+\varepsilon_{H1}\right](1-T_{2}/T_{H})(1+T_{0}/T_{H})-k\varepsilon_{L1}[\left(T_{5}/T_{H}-T_{L}/T_{H}\right)\\ +(T_{5}/T_{L}-1)(T_{0}/T_{H})] \end{array}}{\begin{array}{l} {\left[\left(1-\varepsilon_{L1})(T_{5}/T_{2})+\varepsilon_{L1}(T_{L}/T_{2}\right)\right]}^{k-1}\cdot \left\{(1+b)y{\left[\left(1-\varepsilon_{L1})+\varepsilon_{L1}(T_{L}/T_{5}\right)\right]}^{-1}-(1+b)y+1\right\}\\ +\left[(1-\varepsilon_{H2})+\frac{\varepsilon_{H2}}{\varepsilon_{H1}+(1-\varepsilon_{H1})(T_{2}/T_{H})}\right]\times \{\{(1-\varepsilon_{H1})(1-\varepsilon_{H2})\cdot {\left(\frac{T_{5}}{T_{2}}\right)}^{-1}+[(1-\varepsilon_{H2})\varepsilon_{H1}\\ {+\varepsilon_{H2}]\cdot {\left(T_{5}/T_{H}\right)}^{-1}\}}^{\frac{1}{k-1}}+(1+a)x-1\}-\left[(1+a)x+1\right] \end{array}}$$
(4) When $T_{6}=T_{1}$, i.e., $\varepsilon_{L1}=0$, Equations (19), (28) and (32) are transformed into expressions of η, $\bar{P}$ and $\bar{E}$ of an endoreversible DDC with finite speed of the piston and finite rate of HT:
  (42) $$\eta_{DDC}=1-\frac{\varepsilon_{L2}(T_{5}/T_{H}-T_{L}/T_{H})}{\left[k\varepsilon_{H2}(1-\varepsilon_{H1})+\varepsilon_{H1}\right](1-T_{2}/T_{H})}$$
  (43) $${\bar{P}}_{DDC}=\frac{\left[k\varepsilon_{H2}(1-\varepsilon_{H1})+\varepsilon_{H1}\right](1-T_{2}/T_{H})-\varepsilon_{L2}(T_{5}/T_{H}-T_{L}/T_{H})}{\begin{array}{l} {\left[\left(1-\varepsilon_{L2})(T_{5}/T_{2})+\varepsilon_{L2}(T_{L}/T_{2}\right)\right]}^{k-1}+\left\{(1-\varepsilon_{H2})+\varepsilon_{H2}/\left[\varepsilon_{H1}+(1-\varepsilon_{H1})(T_{2}/T_{H})\right]\right\}\\ \times \left\{{\left\{(1-\varepsilon_{H1})(1-\varepsilon_{H2})\cdot {\left(T_{5}/T_{2}\right)}^{-1}+\left[(1-\varepsilon_{H2})\varepsilon_{H1}+\varepsilon_{H2}\right]\cdot {\left(T_{5}/T_{H}\right)}^{-1}\right\}}^{\frac{1}{k-1}}+(1+a)x-1\right\}\\ -\left[(1+a)x+1\right] \end{array}}$$
  (44) $${\bar{E}}_{DDC}=\frac{\begin{array}{l} \left[k\varepsilon_{H2}(1-\varepsilon_{H1})+\varepsilon_{H1}\right](1-T_{2}/T_{H})(1+T_{0}/T_{H})-\varepsilon_{L2}[\left(T_{5}/T_{H}-T_{L}/T_{H}\right)\\ +(T_{5}/T_{L}-1)(T_{0}/T_{H})] \end{array}}{\begin{array}{l} {\left[\left(1-\varepsilon_{L2})(T_{5}/T_{2})+\varepsilon_{L2}(T_{L}/T_{2}\right)\right]}^{k-1}+\left\{(1-\varepsilon_{H2})+\varepsilon_{H2}/\left[\varepsilon_{H1}+(1-\varepsilon_{H1})(T_{2}/T_{H})\right]\right\}\\ \times \left\{{\left\{(1-\varepsilon_{H1})(1-\varepsilon_{H2})\cdot {\left(T_{5}/T_{2}\right)}^{-1}+\left[(1-\varepsilon_{H2})\varepsilon_{H1}+\varepsilon_{H2}\right]\cdot {\left(T_{5}/T_{H}\right)}^{-1}\right\}}^{\frac{1}{k-1}}+(1+a)x-1\right\}\\ -\left[(1+a)x+1\right] \end{array}}$$
(5) When $T_{2}=T_{3}$ and $T_{5}=T_{6}$, i.e., $\varepsilon_{H1}=0$ and $\varepsilon_{L2}=0$, Equations (19), (28) and (32) are transformed into expressions of η, $\bar{P}$ and $\bar{E}$ of an endoreversible Diesel-Atkinson cycle with finite speed of the piston and finite rate of HT:
  (45) $$\eta_{Diesel-Atkinson\;cycle}=1-\frac{\varepsilon_{L1}(T_{5}/T_{H}-T_{L}/T_{H})}{\varepsilon_{H2}(1-T_{2}/T_{H})}$$
  (46) $${\bar{P}}_{Diesel-Atkinson\;cycle}=\frac{k\varepsilon_{H2}(1-T_{2}/T_{H})-k\varepsilon_{L1}(T_{5}/T_{H}-T_{L}/T_{H})}{\begin{array}{l} {\left[\left(1-\varepsilon_{L1})(T_{5}/T_{2})+\varepsilon_{L1}(T_{L}/T_{2}\right)\right]}^{k-1}\cdot \{(1+b)y{\left[\left(1-\varepsilon_{L1})+\varepsilon_{L1}(T_{L}/T_{5}\right)\right]}^{-1}\\ -(1+b)y+1\}+\left[(1-\varepsilon_{H2})+\varepsilon_{H2}\cdot {\left(T_{2}/T_{H}\right)}^{-1}\right]\times \{[(1-\varepsilon_{H2})\cdot {\left(T_{5}/T_{2}\right)}^{-1}\\ {+\varepsilon_{H2}\cdot {\left(T_{5}/T_{H}\right)}^{-1}]}^{\frac{1}{k-1}}+(1+a)x-1\}-\left[(1+a)x+1\right] \end{array}}$$
  (47) $${\bar{E}}_{Diesel-Atkinson\;cycle}=\frac{\begin{array}{l} k\varepsilon_{H2}(1-T_{2}/T_{H})(1+T_{0}/T_{H})-k\varepsilon_{L1}[\left(T_{5}/T_{H}-T_{L}/T_{H}\right)\\ +(T_{5}/T_{L}-1)(T_{0}/T_{H})] \end{array}}{\begin{array}{l} {\left[\left(1-\varepsilon_{L1})(T_{5}/T_{2})+\varepsilon_{L1}(T_{L}/T_{2}\right)\right]}^{k-1}\cdot \{(1+b)y{\left[\left(1-\varepsilon_{L1})+\varepsilon_{L1}(T_{L}/T_{5}\right)\right]}^{-1}\\ -(1+b)y+1\}+\left[(1-\varepsilon_{H2})+\varepsilon_{H2}\cdot {\left(T_{2}/T_{H}\right)}^{-1}\right]\times \{[(1-\varepsilon_{H2})\cdot {\left(T_{5}/T_{2}\right)}^{-1}\\ {+\varepsilon_{H2}\cdot {\left(T_{5}/T_{H}\right)}^{-1}]}^{\frac{1}{k-1}}+(1+a)x-1\}-\left[(1+a)x+1\right] \end{array}}$$
(6) When $T_{2}=T_{3}$ and $T_{6}=T_{1}$, i.e., $\varepsilon_{H1}=0$ and $\varepsilon_{L1}=0$, Equations (19), (28) and (32) are transformed into expressions of η, $\bar{P}$ and $\bar{E}$ of an endoreversible DC with finite speed of the piston and finite rate of HT:
  (48) $$\eta_{DC}=1-\frac{\varepsilon_{L2}(T_{5}/T_{H}-T_{L}/T_{H})}{k\varepsilon_{H2}(1-T_{2}/T_{H})}$$
  (49) $${\bar{P}}_{DC}=\frac{k\varepsilon_{H2}(1-T_{2}/T_{H})-\varepsilon_{L2}(T_{5}/T_{H}-T_{L}/T_{H})}{\begin{array}{l} {\left[\left(1-\varepsilon_{L2})(T_{5}/T_{2})+\varepsilon_{L2}(T_{L}/T_{2}\right)\right]}^{k-1}+\left[(1-\varepsilon_{H2})+\varepsilon_{H2}\cdot {\left(T_{2}/T_{H}\right)}^{-1}\right]\\ \times \left\{{\left[(1-\varepsilon_{H2})\cdot {\left(T_{5}/T_{2}\right)}^{-1}+\varepsilon_{H2}\cdot {\left(T_{5}/T_{H}\right)}^{-1}\right]}^{\frac{1}{k-1}}+(1+a)x-1\right\}-\left[(1+a)x+1\right] \end{array}}$$
  (50) $${\bar{E}}_{DC}=\frac{k\varepsilon_{H2}(1-T_{2}/T_{H})(1+T_{0}/T_{H})-\varepsilon_{L2}[\left(T_{5}/T_{H}-T_{L}/T_{H}\right)+(T_{5}/T_{L}-1)(T_{0}/T_{H})]}{\begin{array}{l} {\left[\left(1-\varepsilon_{L2})(T_{5}/T_{2})+\varepsilon_{L2}(T_{L}/T_{2}\right)\right]}^{k-1}+\left[(1-\varepsilon_{H2})+\varepsilon_{H2}\cdot {\left(T_{2}/T_{H}\right)}^{-1}\right]\\ \times \left\{{\left[(1-\varepsilon_{H2})\cdot {\left(T_{5}/T_{2}\right)}^{-1}+\varepsilon_{H2}\cdot {\left(T_{5}/T_{H}\right)}^{-1}\right]}^{\frac{1}{k-1}}+(1+a)x-1\right\}-\left[(1+a)x+1\right] \end{array}}$$
(7) When $T_{3}=T_{4}$ and $T_{5}=T_{6}$, i.e., $\varepsilon_{H2}=0$ and $\varepsilon_{L2}=0$, Equations (19), (28) and (32) are transformed into expressions of η, $\bar{P}$ and $\bar{E}$ of an endoreversible Otto-Atkinson cycle with finite speed of the piston and finite rate of HT:
  (51) $$\eta_{Otto-Atkinson\;cycle}=1-\frac{k\varepsilon_{L1}(T_{5}/T_{H}-T_{L}/T_{H})}{\varepsilon_{H1}(1-T_{2}/T_{H})}$$
  (52) $${\bar{P}}_{Otto-Atkinson\;cycle}=\frac{\varepsilon_{H1}(1-T_{2}/T_{H})-k\varepsilon_{L1}(T_{5}/T_{H}-T_{L}/T_{H})}{\begin{array}{l} {\left[\left(1-\varepsilon_{L1})(T_{5}/T_{2})+\varepsilon_{L1}(T_{L}/T_{2}\right)\right]}^{k-1}\cdot \{(1+b)y{\left[\left(1-\varepsilon_{L1})+\varepsilon_{L1}(T_{L}/T_{5}\right)\right]}^{-1}\\ -(1+b)y+1\}+{\left[(1-\varepsilon_{H1})\cdot {\left(T_{5}/T_{2}\right)}^{-1}+\varepsilon_{H1}\cdot {\left(T_{5}/T_{H}\right)}^{-1}\right]}^{\frac{1}{k-1}}-2 \end{array}}$$
  (53) $${\bar{E}}_{Otto-Atkinson\;cycle}=\frac{\begin{array}{l} \varepsilon_{H1}(1-T_{2}/T_{H})(1+T_{0}/T_{H})-k\varepsilon_{L1}[\left(T_{5}/T_{H}-T_{L}/T_{H}\right)\\ +(T_{5}/T_{L}-1)(T_{0}/T_{H})] \end{array}}{\begin{array}{l} {\left[\left(1-\varepsilon_{L1})(T_{5}/T_{2})+\varepsilon_{L1}(T_{L}/T_{2}\right)\right]}^{k-1}\cdot \{(1+b)y{\left[\left(1-\varepsilon_{L1})+\varepsilon_{L1}(T_{L}/T_{5}\right)\right]}^{-1}\\ -(1+b)y+1\}+{\left[(1-\varepsilon_{H1})\cdot {\left(T_{5}/T_{2}\right)}^{-1}+\varepsilon_{H1}\cdot {\left(T_{5}/T_{H}\right)}^{-1}\right]}^{\frac{1}{k-1}}-2 \end{array}}$$
(8) When $T_{3}=T_{4}$ and $T_{6}=T_{1}$, i.e., $\varepsilon_{H2}=0$ and $\varepsilon_{L1}=0$, Equations (19), (28) and (32) are transformed into expressions of η, $\bar{P}$ and $\bar{E}$ of an endoreversible OC with finite speed of the piston and finite rate of HT:
  (54) $$\eta_{OC}=1-\frac{\varepsilon_{L2}(T_{5}/T_{H}-T_{L}/T_{H})}{\varepsilon_{H1}(1-T_{2}/T_{H})}$$
  (55) $${\bar{P}}_{OC}=\frac{\varepsilon_{H1}(1-T_{2}/T_{H})-\varepsilon_{L2}(T_{5}/T_{H}-T_{L}/T_{H})}{{\left[\left(1-\varepsilon_{L2})(T_{5}/T_{2})+\varepsilon_{L2}(T_{L}/T_{2}\right)\right]}^{k-1}+[(1-\varepsilon_{H1})\cdot {\left(T_{5}/T_{2}\right)}^{-1}{+\varepsilon_{H1}\cdot {\left(T_{5}/T_{H}\right)}^{-1}]}^{\frac{1}{k-1}}-2}$$
  (56) $${\bar{E}}_{OC}=\frac{\varepsilon_{H1}(1-T_{2}/T_{H})(1+T_{0}/T_{H})-\varepsilon_{L2}[\left(T_{5}/T_{H}-T_{L}/T_{H}\right)+(T_{5}/T_{L}-1)(T_{0}/T_{H})]}{{\left[\left(1-\varepsilon_{L2})(T_{5}/T_{2})+\varepsilon_{L2}(T_{L}/T_{2}\right)\right]}^{k-1}+[(1-\varepsilon_{H1})\cdot {\left(T_{5}/T_{2}\right)}^{-1}{+\varepsilon_{H1}\cdot {\left(T_{5}/T_{H}\right)}^{-1}]}^{\frac{1}{k-1}}-2}$$

## 4. Numerical Examples

When the value of ρ (or $r_{M}$) is set, $T_{2}$ (or $T_{5}$) can be calculated by combining with Equations (10) and (11) (or Equations (9) and (12)). Then, $T_{1}$, $T_{3}$, $T_{4}$, $T_{6}$ and $T_{5}$ (or $T_{2}$) can be obtained according to Equations (9)–(12) and (16) (or Equation (17)). Substituting temperatures into Equations (19), (28) and (32), η, $\bar{P}$ and $\bar{E}$ can be obtained.

According to Refs. [68,90,93], it is set that $T_{0}=T_{L}=300\;K$, $T_{H}=800\;K$, k=1.4, $\varepsilon_{H1}=\varepsilon_{H2}=\varepsilon_{L1}=\varepsilon_{L2}=0.25$, ρ=1~1.2, x=1,10,100, y=1,10,100, a=1,3,6 and b=1,3,6 in the calculations. Thus, the characteristics relationships between $\bar{P}$ and ρ, between $\bar{P}$ and η, as well as between $\bar{E}$ and ρ of an endoreversible DMC with finite speed of the piston can be obtained.

### 4.1. Effects of x and y on $\bar{P}$, η and $\bar{E}$

**Figure 2:**
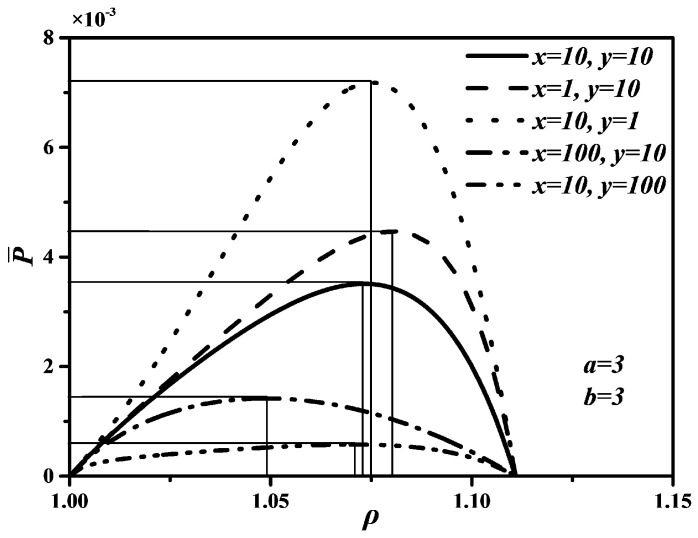
Effects of x and y on $\bar{P}$ versus ρ characteristic.

**Figure 3:**
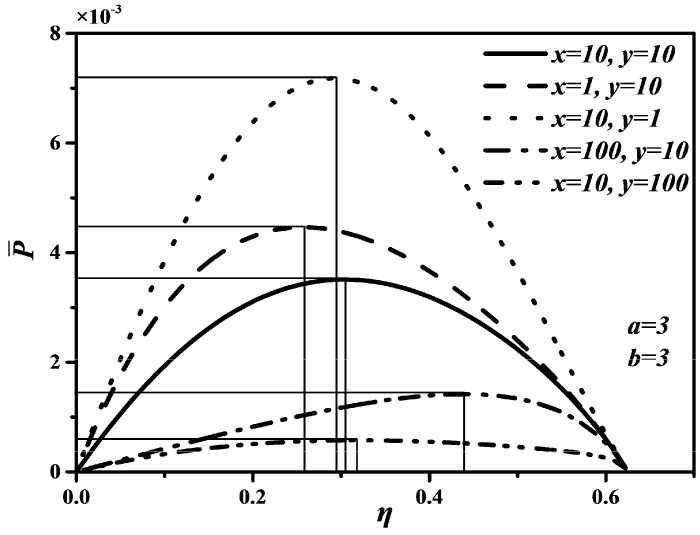
Effects of x and y on $\bar{P}$ versus η characteristic.

**Figure 4:**
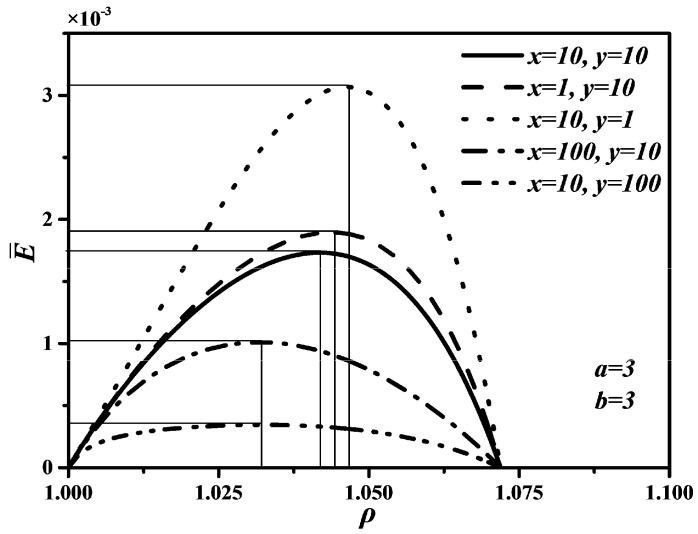
Effects of x and y on $\bar{E}$ versus ρ characteristic.

Figure 2, Figure 3 and Figure 4 show the non-dimensional power output ($\bar{P}$) versus cut-off ratio (ρ), $\bar{P}$ versus thermal efficiency (η), and non-dimensional ecological function ($\bar{E}$) versus ρ characteristics when x and y take different values, respectively. In Figure 2 and Figure 4, one can see that $\bar{P}$ and $\bar{E}$ first increase and then start to decrease with increasing ρ. There are two different ρ when $\bar{P}$ and $\bar{E}$ reach to zeroes, respectively. Moreover, there are different optimal cut-off ratios ($\rho_{opt}$) to make $\bar{P}$ and $\bar{E}$ reach their maximum values, respectively. However, there are some differences between the curves of $\bar{P}$ versus ρ and $\bar{E}$ versus ρ. For example, the ending point of ρ is 1.111 corresponding to $\bar{P}=0$, but the ending point of ρ is 1.071 corresponding to $\bar{E}=0$. The reason is $\bar{P}$ should be larger than zero if $\bar{E}=0$ according to the definition of $E=P-T_{0}\sigma$, which shrinks the range of ρ.

In Figure 2 and Figure 4, it is obvious that the larger x and y are, the smaller the maximum values of $\bar{P}$ and $\bar{E}$ are. That is, the faster piston speeds in processes 3→4 and 6→1 are, the larger the maximum values of $\bar{P}$ and $\bar{E}$ are. Further, as x and y increase, the optimal $\rho_{opt_{\bar{P}}}$ corresponding to the maximum $\bar{P}$ and optimal $\rho_{opt_{\bar{E}}}$ corresponding to the maximum $\bar{E}$ will decrease, but the variation range of the optimal $\rho_{opt_{\bar{P}}}$ with x is larger than that of the optimal $\rho_{opt_{\bar{P}}}$ with y, the variation range of the optimal $\rho_{opt_{\bar{E}}}$ with y is larger than that of the optimal $\rho_{opt_{\bar{E}}}$ with x. The effects of y on $\bar{P}$ and $\bar{E}$ are more significant than those of x, which means the piston speed in constant pressure heat rejection process has more marked impact on $\bar{P}$ and $\bar{E}$ than in constant pressure heat addition process. It can be concluded from Figure 3 that x and y have no effect on η. The maximum η is equal to 0.622 whatever values x and y take. The reason is that W and $Q_{H}$ are uncorrelated with cycle time τ, leading to η be uncorrelated with x and y according to the definition of $\eta =W/Q_{H}$. Moreover, as x and y increase, the thermal efficiency ($\eta_{\bar{P}}$) at the maximum $\bar{P}$ will increase.

### 4.2. Effects of a and b on $\bar{P}$, η and $\bar{E}$

**Figure 5:**
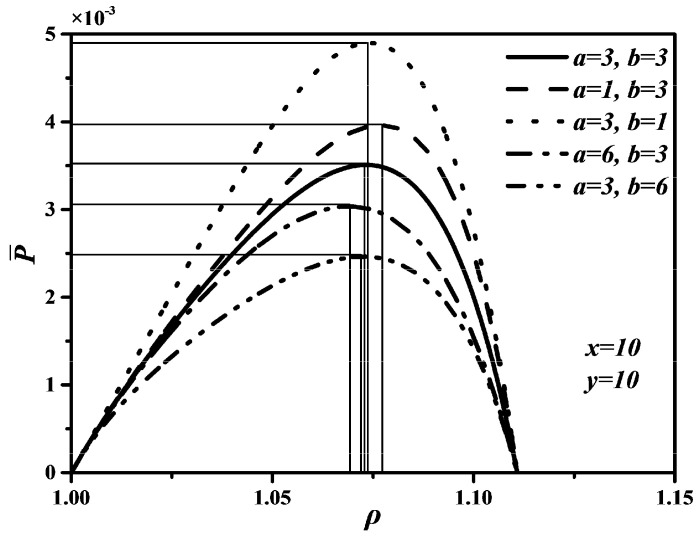
Effects of a and b on $\bar{P}$ versus ρ characteristic.

**Figure 6:**
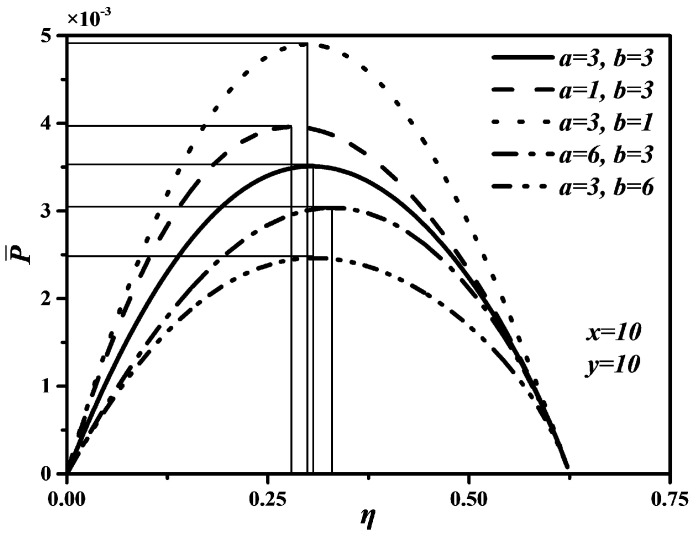
Effects of a and b on $\bar{P}$ versus η characteristic.

**Figure 7:**
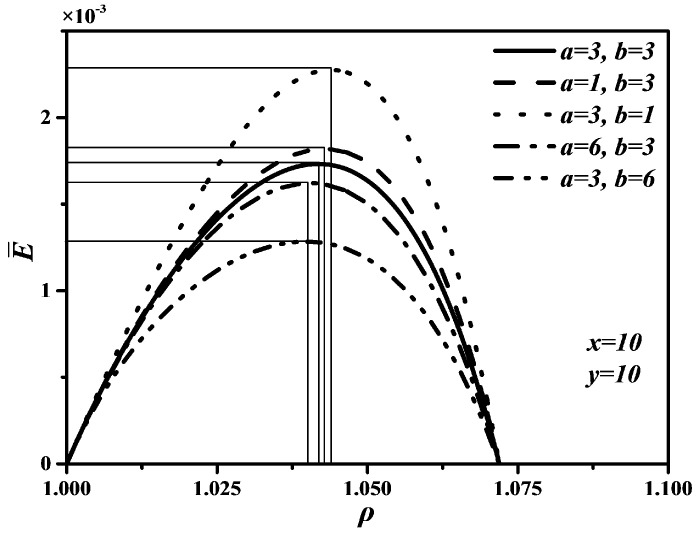
Effects of a and b on $\bar{E}$ versus ρ characteristic.

Figure 5, Figure 6 and Figure 7 show $\bar{P}$ versus ρ, $\bar{P}$ versus η, and $\bar{E}$ versus ρ characteristics when a and b take different values, which are similar to curves of $\bar{P}$ versus ρ, $\bar{P}$ versus η, and $\bar{E}$ versus ρ characteristics under different x and y. In Figure 5 and Figure 7, one can see that the smaller a and b are, the larger the maximum values of $\bar{P}$ and $\bar{E}$ are. That is, the shorter the times in processes 2→3 and 5→6 are, the larger the maximum values of $\bar{P}$ and $\bar{E}$ are. Further, as a and b increase, the optimal $\rho_{opt_{\bar{P}}}$ and optimal $\rho_{opt_{\bar{E}}}$ will decrease, but the variation range of the optimal $\rho_{opt_{\bar{P}}}$ with a is larger than that of the optimal $\rho_{opt_{\bar{P}}}$ with b, the variation range of the optimal $\rho_{opt_{\bar{E}}}$ with b is larger than that of the optimal $\rho_{opt_{\bar{E}}}$ with a. The effects of b on $\bar{P}$ and $\bar{E}$ are more significant than those of a, which means the piston speed in constant volume heat rejection process has more marked impact on $\bar{P}$ and $\bar{E}$ than that in constant volume heat addition process. It can be concluded from Figure 6 that a and b have no effect on η. The maximum η is equal to 0.622 whatever values a and b take. The reason is as same as that of η being uncorrelated with x and y. Moreover, as a and b increase, the thermal efficiency ($\eta_{\bar{P}}$) at the maximum $\bar{P}$ will increase slightly.

### 4.3. Performance Comparison

**Figure 8:**
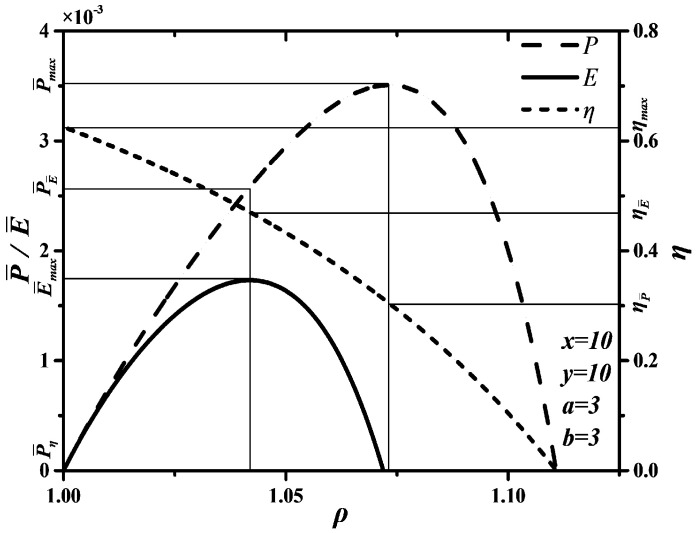
$\bar{P}$, η and $\bar{E}$ characteristics of the Dual-Miller cycle.

Figure 8 gives the curves between $\bar{P}$ and ρ, between η and ρ, as well as between $\bar{E}$ and ρ. When $\bar{P}$, η and $\bar{E}$ are chosen as optimization objective functions, the DMC engines will be in different conditions. According to Figure 8, the following can be concluded.

(1) When $\bar{P}$ is chosen as optimization objective function, the maximum $\bar{P}$ is ${\bar{P}}_{\max}=0.0035$, η corresponding to the maximum $\bar{P}$ is $\eta_{\bar{P}}=0.306$. However, the maximum η is $\eta_{\max}=0.622$, $\eta_{\bar{P}}/\eta_{\max}=0.492$, so choosing $\bar{P}$ as optimization objective function sacrifices most of η.
(2) When η is chosen as optimization objective function, $\bar{P}$ corresponding to the maximum η is ${\bar{P}}_{\eta }=0$, ${\bar{P}}_{\eta }/{\bar{P}}_{\max}=0$. Although η can reach the maximum, $\bar{P}$ is zero when optimizing η. Thus, choosing η as optimization objective function is unreasonable.
(3) When $\bar{E}$ is chosen as optimization objective function, the maximum $\bar{E}$ is ${\bar{E}}_{\max}=0.0017$, $\bar{P}$ corresponding to the maximum $\bar{E}$ is ${\bar{P}}_{\bar{E}}=0.0026$, ${\bar{P}}_{\bar{E}}/{\bar{P}}_{\max}=0.743$, η corresponding to the maximum $\bar{E}$ is $\eta_{\bar{E}}=0.470$, $\eta_{\bar{E}}/\eta_{\max}=0.756$. Thus, choosing $\bar{E}$ as optimization objective function can improve η compared with choosing $\bar{P}$ as optimization objective function and can also improve $\bar{P}$ compared with choosing η as optimization objective function.
(4) There are ${\bar{P}}_{\eta }<{\bar{P}}_{\bar{E}}<{\bar{P}}_{\max}$ and $\eta_{\bar{P}}<\eta_{\bar{E}}<\eta_{\max}$. When $\bar{E}$ is chosen as optimization objective function, $\bar{P}$ and η may be smaller than their maxima, but they can reach relatively large values at the same time. Hence, optimizing $\bar{E}$ is the best compromise optimization between optimizing $\bar{P}$ and optimizing η.

### 4.4. Corollary 75–25

In thermodynamic (and thermoeconomic) optimization of some endoreversible models of heat engines, there is a reduction of the total entropy production (Corollary 75–25 [98,99]) when the ecological function is maximized in comparison with that obtained under maximum power output. Corollary 75–25 means that the maximization of the ecological function leads to an engine performance with a power output around 75% of the maximum power output and an entropy production rate around 25% of the entropy production rate at maximum power condition [98,99].

**Figure 9:**
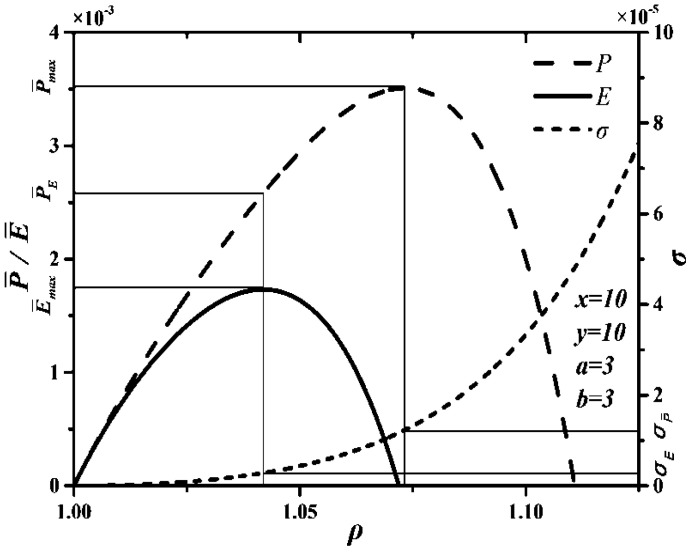
Power output, ecological function and entropy generation rate for the Dual-Miller cycle.

The curves of power output, ecological function and entropy generation rate versus cut-off ratio for the endoreversible Dual-Miller cycle are depicted in Figure 9. The values of maximum power output, power output corresponding to maximum ecological function, entropy generation rate corresponding to maximum power output and entropy generation rate corresponding to maximum ecological function are listed in Table 1. They show that the property of ecological function is also concordant with Corollary 75–25 for the endoreversible Dual-Miller cycle with finite speed of the piston.

## 5. Conclusions

The performances of the non-dimensional power output, thermal efficiency and non-dimensional ecological function of an endoreversible DMC with finite rate of HT are analyzed and optimized based on finite speed of the piston. Performance characteristics are discussed and analyzed when DMC are simplified to other cycles. The effects of ρ and piston speeds on performance of the cycle are examined via numerical examples. The results show that, the faster the piston speeds are, the larger the maximum values of $\bar{P}$ and $\bar{E}$ are. The optimal cut-off ratio $\rho_{opt}$ will increase if piston speeds increase in heat addition processes and heat rejection processes. The effects of time in constant volume heat rejection process on $\bar{P}$ and $\bar{E}$ are more marked than those of time in constant volume heat addition process, the effects of time in constant pressure heat rejection process on $\bar{P}$ and $\bar{E}$ are also more marked than those of time in constant pressure heat addition process. Choosing ecological function as optimization objective function is more significant compared with choosing power output and thermal efficiency as optimization objective functions.

The results obtained herein show that there are three aspects to improve the performance when designing the Dual-Miller cycle engines. Firstly, improving piston speed can increase the power output. Secondly, excessive times in heat addition and heat rejection processes have negative impacts on performance of the cycle engines, thus the times in heat rejection processes should be decreased as much as possible. Lastly, taking ecological function as a design criterion can improve comprehensive performance (power output and thermal efficiency) of the cycle engines.

The cut-off ratio is an important design parameter for Dual-Miller cycle heat engines; it can determine the running forms of the engines. For example, the heat engine can run with maximum power output, maximum thermal efficiency and maximum ecological function conditions. Thus, optimizing the cut-off ratio is necessary, and optimal cut-off ratio is also important to design heat engines with different conditions.

The obtained results herein are based on the assumptions that the working fluid is an ideal gas and its specific heat is constant. The specific heat of the working fluid will change with its temperature in actual heat engines. The variable specific heat characteristic of the working fluid will be considered in the next studies.

## Figures and Tables

**Table 1:** Values of power output and entropy generation rate with different optimal conditions.

Optimal Conditions	Power Output	Entropy Generation Rate
${\bar{E}}_{\max}$	2.63 × 10^−3^	3.013 × 10^−6^
${\bar{P}}_{\max}$	3.51 × 10^−3^	1.222 × 10^−5^
ratio	74.9%	24.7%

## Nomenclatures

a	constant
$A_{ps}$	area of piston face ($m^{2}$)
b	constant
$C_{p}$	specific heat at constant pressure (kJ/(kg⋅K))
$C_{v}$	specific heat at constant volume (kJ/(kg⋅K))
E	ecological function (kW)
ECOP	ecological coefficient of performance
F	area of heat transfer ($m^{2}$)
k	specific heat ratio
L	stroke length (m)
m	mass of working fluid (kg)
N	number of heat transfer unites
P	power output (kW)
Q	quantity of heat transfer (kJ)
S	entropy generation (kJ/K)
T	temperature (K)
u	piston speed (m/s)
U	heat transfer coefficient ($W/(m^{2}\cdot K)$)
V	volume ($m^{3}$)
Greek symbols	
ε	effectiveness of heat exchanger
*η*	thermal efficiency
σ	entropy generation rate (kW/K)
τ	cycle period (s)
Subscripts	
*H*	high temperature heat reservoir
L	low temperature heat reservoir
opt	optimal value
1,2,3,4,5,6	cycle state points
Superscripts	
$\dot{}$	rate
$\bar{}$	non-dimensional

## Abbreviations

The following abbreviations are used in this manuscript:

DAC	Dual-Atkinson cycle
DC	Diesel cycle
DDC	Dual-Diesel cycle
DiMC	Diesel-Miller cycle
DMC	Dual-Miller cycle
FST	finite speed thermodynamics
HEC	heat engine cycle
HL	heat leakage
HT	heat transfer
ICE	internal combustion engine
OC	Otto cycle
OMC	Otto-Miller cycle
TFS	thermodynamics with finite speed

## References

[1] Curzon F.L., Ahlborn B. (1975). Efficiency of a Carnot engine at maximum power output. Am. J. Phys..

[2] Andresen B. (1983). Finite-Time Thermodynamics.

[3] Andresen B., Salamon P., Berry R.S. (1984). Thermodynamics in finite time. Phys. Today.

[4] Bejan A. (1996). Entropy generation on minimization: The new thermodynamics of finite-size device and finite-time processes. J. Appl. Phys..

[5] Chen L.G., Wu C., Sun F.R. (1999). Finite time thermodynamic optimization or entropy generation minimization of energy systems. J. Non-Equilib. Thermodyn..

[6] Chen L.G. (2005). Finite-Time Thermodynamic Analysis of Irreversible Processes and Cycles.

[7] Feidt M. (2009). Optimal Thermodynamics-New Upperbounds. Entropy.

[8] Andresen B. (2011). Current trends in finite-time thermodynamics. Angew. Chem. Int. Ed..

[9] Quijano J., Lin H. (2014). Entropy in the critical zone: A comprehensive review. Entropy.

[10] Feng H.J., Chen L.G., Xie Z.H., Sun F.R. (2016). Constructal design for a rectangular body with nonuniform heat generation. Eur. Phys. J. Plus.

[11] Feidt M. (2017). The history and perspectives of efficiency at maximum power of the Carnot engine. Entropy.

[12] Honig J. (2013). On the entropy of a class of irreversible processes. Entropy.

[13] Chen L.G., Feng H.J., Xie Z.H. (2016). Generalized thermodynamic optimization for iron and steel production processes: Theoretical exploration and application cases. Entropy.

[14] Chen L.G., Xia S.J. (2017). Generalized Thermodynamic Dynamic-Optimization for Irreversible Processes.

[15] Acikkalp E. (2013). Models for optimum thermo-ecological criteria of actual thermal cycles. Therm. Sci..

[16] Açıkkalp E., Yamik H. (2013). Limits and optimization of power input or output of actual thermal cycles. Entropy.

[17] Açıkkalp E. (2015). Methods used for evaluation of actual power generating thermal cycles and comparing them. Int. J. Electr. Power Energy Syst..

[18] Chen L.G., Xia S.J. (2017). Generalized Thermodynamic Dynamic-Optimization for Irreversible Cycles: Thermodynamic and Chemical Theoretical Cycles.

[19] Chen L.G., Xia S.J. (2017). Generalized Thermodynamic Dynamic-Optimization for Irreversible Cycles: Engineering Thermodynamic Plants and Generalized Engine Cycles.

[20] Reyes-Ramírez I., Barranco-Jiménez M.A., Rojas-Pacheco A., Guzmán-Vargas L. (2014). Global stability analysis of a Curzon-Ahlborn heat engine using the Lyapunov method. Phys. A Stat. Mech. Appl..

[21] Açıkkalp E., Yamik H. (2015). Modeling and optimization of maximum available work for irreversible gas power cycles with temperature dependent specific heat. J. Non-Equilib. Thermodyn..

[22] Xia S.J., Chen L.G., Sun F.R. (2016). Maximum cycle work output optimization for generalized radiative law Otto cycle engines. Eur. Phys. J. Plus.

[23] Qin X.Y., Chen L.G., Ge Y.L., Sun F.R. (2015). Thermodynamic modeling and performance analysis of variable-temperature heat reservoir absorption heat pump cycle. Phys. A Stat. Mech. Appl..

[24] Zhang L., Chen L.G., Sun F.R. (2016). Power optimization chemically driven heat engine based on first and second order reaction kinetic theory and probability theory. Phys. A Stat. Mech. Appl..

[25] Kosloff R. (2013). Quantum thermodynamics: A dynamical viewpoint. Entropy.

[26] Von Spakovsky M.R., Gemmer J. (2014). Some trends in quantum thermodynamics. Entropy.

[27] Açıkkalp E., Caner N. (2015). Determining of the optimum performance of a nano scale irreversible Dual cycle with quantum gases as working fluid by using different methods. Phys. A Stat. Mech. Appl..

[28] Açıkkalp E., Caner N. (2015). Determining performance of an irreversible nano scale dual cycle operating with Maxwell-Boltzmann gas. Phys. A Stat. Mech. Appl..

[29] Açıkkalp E., Caner N. (2015). Application of exergetic sustainable index to the quantum irreversible Diesel refrigerator cycles for 1D box system. Eur. Phys. J. Plus.

[30] Ahmadi M.H., Ahmadi M.A., Pourfayaz F. (2015). Performance assessment and optimization of an irreversible nano-scale Stirling engine cycle operating with Maxwell-Boltzmann gas. Eur. Phys. J. Plus.

[31] Kosloff R., Rezek Y. (2017). The quantum harmonic Otto cycle. Entropy.

[32] Ding Z.M., Chen L.G., Wang W.H., Ge Y.L., Sun F.R. (2015). Exploring the operation of a microscopic energy selective electron engine. Phys. A Stat. Mech. Appl..

[33] Ding Z.M., Chen L.G., Ge Y.L., Sun F.R. (2016). Performance optimization of total momentum filtering double-resonance energy selective electron heat pump. Phys. A Stat. Mech. Appl..

[34] Zhou J.L., Chen L.G., Ding Z.M., Sun F.R. (2016). Exploring the optimal performance of irreversible single resonance energy selective electron refrigerator. Eur. Phys. J. Plus.

[35] Ge Y.L., Chen L.G., Sun F.R. (2016). Progress in finite time thermodynamic studies for internal combustion engine cycles. Entropy.

[36] Sun F.R., Chen L.G., Chen W.Z. (1989). Finite time Thermodynamics analysis and evaluation for a heat engine with steady-state energy conversion between heat sources. J. Eng. Therm. Energy Power.

[37] Wu C. (1990). Specific power optimization of a closed-cycle OTEC plants. Ocean Eng..

[38] Sahin B., Kodal A., Yavuz H. (1996). Maximum power density analysis of an endoreversible Carnot heat engine. Energy.

[39] Sahin B., Kodal A., Yavuz H. (1995). Efficiency of Joule-Brayton engine at maximum power density. J. Phys. D Appl. Phys..

[40] Jubeh N.M. (2005). Exergy analysis and second law efficiency of a regenerative Brayton cycle with isothermal heat addition. Entropy.

[41] Labrecque R. (2009). Exergy as a useful variable for quickly assessing the theoretical maximum power of salinity gradient energy systems. Entropy.

[42] Açıkkalp E., Caner N. (2015). Performance assessment of an irreversible nano Brayton cycle operating with Maxwell-Boltzmann gas. Eur. Phys. J. Plus.

[43] Açıkkalp E. (2015). Exergetic sustainability evaluation of irreversible Carnot refrigerator. Phys. A Stat. Mech. Appl..

[44] Chen L.G., Sun F.R., Chen W.Z. (1991). Finite time exergoeconomic performance bound and optimization criteria for two-heat-reservoir heat engine. Chin. Sci. Bull..

[45] Wu C., Chen L.G., Sun F.R. (1996). Effect of heat transfer law on finite-time exergoeconomic performance of heat engines. Energy.

[46] Angulo-Brown F. (1991). An ecological optimization criterion for finite-time heat engines. J. Appl. Phys..

[47] Yan Z.J. (1993). Comment on “ecological optimization criterion for finite-time heat engines”. J. Appl. Phys..

[48] Chen L.G., Sun F.R., Chen W.Z. (1994). The ecological figures of merit for thermodynamic cycles. J. Eng. Therm. Energy Power.

[49] Barranco-Jiménez M.A., Chimal-Eguía J.C., Angulo-Brown F. (2006). The Gordon and Zarmi model for convective atmospheric cells under the ecological criterion applied to the planets of the solar system. Rev. Mex. Fís..

[50] Barranco-Jimenez M.A., Angulo-Brown F. (2007). Thermo-economic optimization of a Novikov power plant model under maximum ecological conditions. J. Energy Inst..

[51] Arias-Hernandez L.A., Barranco-Jimenez M.A., Angulo-Brown F. (2009). Comparative analysis of two ecological type modes of performance for a simple energy converter. J. Energy Inst..

[52] Angulo-Brown F., Fernandez-Betanzos J., Diaz-Pico C.A. (1994). Compression ratio of an optimized Otto cycle model. Eur. J. Phys..

[53] Ust Y., Safa A., Sahin B. (2005). Ecological performance analysis of an endoreversible regenerative Brayton heat-engine. Appl. Energy.

[54] Ust Y., Sahin B., Kodal A. (2005). Ecological coefficient of performance (ECOP) optimization for generalized irreversible Carnot heat engines. J. Energy Inst..

[55] Barranco-Jiménez M.A., Angulo-Brown F. (2007). Thermoeconomic optimisation of endoreversible heat engine under maximum modified ecological criterion. J. Energy Inst..

[56] Moscato A.L.S., del Rio Oliveira S. (2015). Net power optimization of an irreversible Otto cycle using ECOP and ecological function. Int. Rev. Mech. Eng..

[57] Gonca G., Sahin B. (2014). Performance optimization of an air-standard irreversible Dual-Atkinson cycle engine based on the ecological coefficient of performance criterion. Sci. World J..

[58] Long R., Liu W. (2015). Ecological optimization for general heat engines. Phys. A Stat. Mech. Appl..

[59] Chen L.G., Zhang W.L., Sun F.R. (2007). Power, efficiency, entropy-generation rate and ecological optimization for a class of generalized irreversible universal heat-engine cycles. Appl. Energy.

[60] Ge Y.L. (2011). Finite Time Thermodynamic Analysis and Optimization for Irreversible Internal Combustion Engine Cycles. Ph.D. Thesis.

[61] Chen L.G., Zhu X.Q., Sun F.R., Wu C. (2005). Ecological optimization for generalized irreversible Carnot refrigerators. J. Phys. D Appl. Phys..

[62] Wu X.H., Chen L.G., Sun F.R. (2015). Local stability of a non-endoreversible Carnot refrigerator working at the maximum ecological function. Appl. Math. Model..

[63] Long R., Liu W. (2016). Ecological optimization and coefficient of performance bounds of general refrigerators. Phys. A Stat. Mech. Appl..

[64] Liu X.W., Chen L.G., Wu F., Sun F.R. (2010). Ecological optimization of an irreversible quantum Carnot heat engine with spin-1/2 systems. Phys. Scripta.

[65] Chen L.G., Kan X.X., Sun F.R., Wu F., Guo F.Z. (2010). Ecological performance optimization of a thermoacoustic heat engine. Rev. Mex. Fis..

[66] Chen L.G., Xia D., Sun F.R. (2010). Ecological optimization of generalized irreversible chemical engines. Int. J. Chem. React. Eng..

[67] Wang H., Wu G.X. (2013). Ecological optimization for generalized irreversible macro/nano thermosize engine. J. Appl. Phys..

[68] Ma K., Chen L.G., Sun F.R. (2013). Ecological performance improved by controlling piston motion: Linear phenomenological system bimolecular, light-driven engine. J. Energy Inst..

[69] Zhou J.L., Chen L.G., Ding Z.M., Sun F.R. (2016). Analysis and optimization with ecological objective function of irreversible single resonance energy selective electron heat engines. Energy.

[70] Long R., Li B.D., Liu Z.C., Liu W. (2016). Ecological analysis of a thermally regenerative electro-chemical cycle. Energy.

[71] Ramírez-Moreno M.A., Angulo-Brown F. (2017). Ecological optimization of a family of *n*-Müser engines for an arbitrary value of the solar concentration factor. Phys. A Stat. Mech. Appl..

[72] Qin X.Y., Chen L.G., Sun F.R. (2003). The universal power and efficiency characteristics for irreversible reciprocating heat engine cycles. Eur. J. Phys..

[73] Ge Y.L., Chen L.G., Sun F.R. (2005). Reciprocating heat-engine cycles. Appl. Energy.

[74] Agrawal D.C., Menon V.J. (2009). Power of a finite speed Carnot engine. Eur. J. Phys..

[75] Agrawal D.C. (2009). A finite speed Curzon-Ahlborn engine. Eur. J. Phys..

[76] Petrescu S., Cristea A.F., Boriaru N., Costea M. Optimization of the irreversible Otto cycle using finite speed thermodynamics and the direct method. Proceedings of the 10th WSEAS International Conference on Mathematical and Computational Methods Science and Engineering (MACMESE’08), Computers and Simulation in Modern Science.

[77] Petrescu S., Boriaru N., Costea M. (2009). Optimization of the irreversible Diesel cycle using finite speed thermodynamics and the direct method. Eng. Sci..

[78] Petrescu S., Harman C., Bejan A., Costea M., Dobre C. (2012). Carnot cycle with external and internal irreversibilities analyzed in thermodynamics with finite speed with the direct method. Rev. Termotehnica.

[79] Petrescu S., Harman C., Petre C., Costea M. (2009). Irreversibility generation analysis of reversed cycle Carnot machine by using the finite speed thermodynamics. Rev. Termotehnica.

[80] Petrescu S., Costea M. (2012). Development of Thermodynamics with Finite Speed and Direct Method.

[81] Petrescu S. (2015). Thermodynamics with Finite Speed and Thermodynamics in Finite Time.

[82] Petrescu S., Feidt M., Enache V., Costea M., Stanciu C., Boriaru N. (2015). Unification perspective of finite physical dimensions thermodynamics and finite speed thermodynamics. Int. J. Energy Environ. Eng..

[83] Yang B., Chen L.G., Sun F.R. (2011). Performance analysis and optimization for an endreversible Carnot heat pump cycle with finite speed of the piston. Int. J. Energy Environ..

[84] Feng H.J., Chen L.G., Sun F.R. (2010). Optimal ratios of the piston speeds for a finite speed endoreversible Carnot heat engine cycle. Rev. Mex. Fis..

[85] Feng H.J., Chen L.G., Sun F.R. (2011). Optimal ratios of the piston speeds for a finite speed irreversible Carnot heat engine cycle. Int. J. Sustain. Energy.

[86] Chen L.G., Feng H.J., Sun F.R. (2011). Optimal piston speed ratio analyses for irreversible Carnot refrigerator and heat pump using finite time thermodynamics, finite speed thermodynamics and direct method. J. Energy Inst..

[87] Hosseinzade H., Sayyaadi H., Babaelahi M. (2015). A new closed-form analytical thermal model for simulating Stirling engines based on polytropic-finite speed thermodynamics. Energy Convers. Manag..

[88] Ahmadi M.H., Ahmadi M.A., Pourfayaz F., Bidi M., Hosseinzade H., Feidt M. (2016). Optimization of powered Stirling heat engine with finite speed thermodynamics. Energy Convers. Manag..

[89] Gonca G., Sahin B., Ust Y. (2013). Performance maps for an air-standard irreversible Dual-Miller cycle (DMC) with late inlet valve closing (LIVC) version. Energy.

[90] Ust Y., Arslan F., Ozsari I., Cakir M. (2015). Thermodynamic performance analysis and optimization of DMC (Dual Miller Cycle) cogeneration system by considering exergetic performance coefficient and total exergy output criteria. Energy.

[91] Gonca G., Sahin B., Ust Y. (2015). Investigation of heat transfer influences on performance of air-standard irreversible Dual-Miller cycle. J. Thermophys. Heat Transf..

[92] Gonca G. (2016). Comparative performance analyses of irreversible OMCE (Otto Miller cycle engine)-DiMCE (Diesel Miller cycle engine)-DMCE (Dual Miller cycle engine). Energy.

[93] Gonca G., Sahin B. (2016). Thermo-ecological performance analyses and optimizations of irreversible gas cycle engines. Appl. Therm. Eng..

[94] Wu Z.X., Chen L.G., Ge Y.L., Sun F.R. (2018). Optimization of the power, efficiency and ecological function for an air-standard irreversible Dual-Miller cycle. Front. Energy.

[95] Wu Z.X., Chen L.G., Ge Y.L., Sun F.R. (2018). Thermodynamic optimization for an air-standard irreversible Dual-Miller cycle with linearly variable specific heat ratio of working fluid. Int. J. Heat Mass Transf..

[96] Wu Z.X., Chen L.G., Ge Y.L., Sun F.R. (2017). Power, efficiency, ecological function and ecological coefficient of performance of an irreversible Dual-Miller cycle (DMC) with nonlinear variable specific heat ratio of working fluid. Eur. Phys. J. Plus.

[97] You J., Chen L.G., Wu Z.X., Sun F.R. (2018). Thermodynamic performance of Dual-Miller cycle (DMC) with polytropic processes based on power output, thermal efficiency and ecological function. Sci. China Tech. Sci..

[98] Arias-Hernández L.A., Angulo-Brown F. (2001). Reply to Comment on A general property of endoreversible thermal engines. J. Appl. Phys..

[99] Barranco-Jiménez M.A. (2009). Finite-time thermoeconomic optimization of a non endoreversible heat engine. Rev. Mex. Fis..

